# Synthetic fertilizers alter floral biophysical cues and bumblebee foraging behavior

**DOI:** 10.1093/pnasnexus/pgac230

**Published:** 2022-11-09

**Authors:** Ellard R Hunting, Sam J England, Kuang Koh, Dave A Lawson, Nadja R Brun, Daniel Robert

**Affiliations:** School of Biological Sciences, University of Bristol, Bristol BS8 1TQ, UK; School of Biological Sciences, University of Bristol, Bristol BS8 1TQ, UK; School of Biological Sciences, University of Bristol, Bristol BS8 1TQ, UK; School of Biological Sciences, University of Bristol, Bristol BS8 1TQ, UK; Biology Department, Woods Hole Oceanographic Institution, 86 Water St, Falmouth, MA 02543, USA; School of Biological Sciences, University of Bristol, Bristol BS8 1TQ, UK

**Keywords:** pollination, imidacloprid, neonicotinoid, electrostatics, humidity

## Abstract

The use of agrochemicals is increasingly recognized as interfering with pollination services due to its detrimental effects on pollinators. Compared to the relatively well-studied chemical toxicity of agrochemicals, little is known on how they influence various biophysical floral cues that are used by pollinating insects to identify floral rewards. Here, we show that widely used horticultural and agricultural synthetic fertilizers affect bumblebee foraging behavior by altering a complex set of interlinked biophysical properties of the flower. We provide empirical and model-based evidence that synthetic fertilizers recurrently alter the magnitude and dynamics of floral electrical cues, and that similar responses can be observed with the neonicotinoid pesticide imidacloprid. We show that biophysical responses interact in modifying floral electric fields and that such changes reduce bumblebee foraging, reflecting a perturbation in the sensory events experienced by bees during flower visitation. This unveils a previously unappreciated anthropogenic interference elicited by agrochemicals within the electric landscape that is likely relevant for a wide range of chemicals and organisms that rely on naturally occurring electric fields.

Significance Statement Flowers exhibit morphological and biophysical adaptations that attract pollinators. A recently discovered ability of several insect species to detect and use electric fields surrounding flowers uncovered the biological significance of natural electric fields. Here, we show that commonly applied synthetic fertilizers and the neonicotinoid pesticide imidacloprid alter the biophysical properties of flowers, affecting both the magnitude and the dynamics of floral electrical cues. These changes result in reduced bumblebee foraging, reflecting a perturbation in the sensory events experienced by bees during flower visitation. Linking fertilizer application to an alteration in pollinator behavior reveals the direct interference by human-made chemicals on how an organism can perceive its physical environment and offers insights into potentially wide-spread perturbations of the biologically relevant electrical environment.

## Introduction

Flowers produce a diverse range of cues and attractants to pollinators that collectively promote localization and pollination (1). These cues encompass morphological and physiological adaptations that are relevant over different spatial scales. On a large scale, pollinators use color, sun, and magnetic fields to navigate the landscape (2, 3). On a smaller scale, pollinators use cues such as odor, floral shape, texture, and humidity to identify floral cues and rewards (1, 4, 5). The recently discovered ability of several insects to detect and discriminate static electric fields (E-fields) surrounding flowers has exposed the biological significance of the natural electric landscape (5–9). Floral E-fields arise from the negative bio-electric potential within the flower and positive charges in the atmosphere, including the electrosphere and positively charged insects such as bees (10, 11). Interactions with plants (e.g. pollen transfer, bee visits, and herbivory) result in immediate changes of the electric potential and field structure surrounding the flower, due to electrokinetic responses of the plant (e.g. sap flow) and diffusion of charges in plant tissues (10, 12, 13). Floral E-fields can therefore provide valuable information for pollinators regarding floral rewards.

The widespread use of chemicals in agriculture and horticulture constitutes a pervasive and substantial source of pollution (14, 15). Agrochemicals, in particular pesticides, have been linked to reductions in pollinator abundance and diversity (16–18), and subtle and sub-lethal mechanisms of toxicity are increasingly being recognized as affecting pollinator health and their ability to orient and forage (19–23). Yet aside from chemical toxicity, little is known about the influence of agrochemicals on the mutualistic interaction between plants and pollinators. Foliar applications of agrochemicals are common practice in horticulture and in recent decades gained popularity in agriculture (24). Mixtures of chemicals, in particular fertilizers, are applied dissolved in water using large-scale spray applications. Such spray applications can potentially change the electric properties of flowers in several ways. Many chemicals carry a charge, or electrostatic additives, designed to effectively adhere and enhance exposure to plants (25, 26), and therefore spray applications can increase humidity and alter the conductivity and permittivity of both the plant surface and its surrounding air. Furthermore, chemicals can modify the E-fields around flowers as chemicals have been shown to alter plant electrophysiology (27). Such changes potentially affect the magnitude and dynamics of natural E-fields that are relevant for flower-pollinator interactions.

Spray applications with insecticides and fungicides have indeed been linked to an immediate temporary decline in bee foraging (28, 29), as well as long-term effects on flower visitation (30). This can manifest itself as an immediate behavioral response wherein bumblebees decide not to land while hovering around treated flowers (28, 29; Supplementary Movie, S1). The ability to sense chemical treatment without direct contact with the flower raises the question as to whether agrochemical application can elicit a distortion of floral cues and therewith affect pollinator behavior and pollination. Here, we show that widely used compound fertilizers can temporarily alter bee foraging behavior due to a biophysical alteration of the electrostatic interaction between flowers and bees.

## Results and discussion

**Fig. 1.**
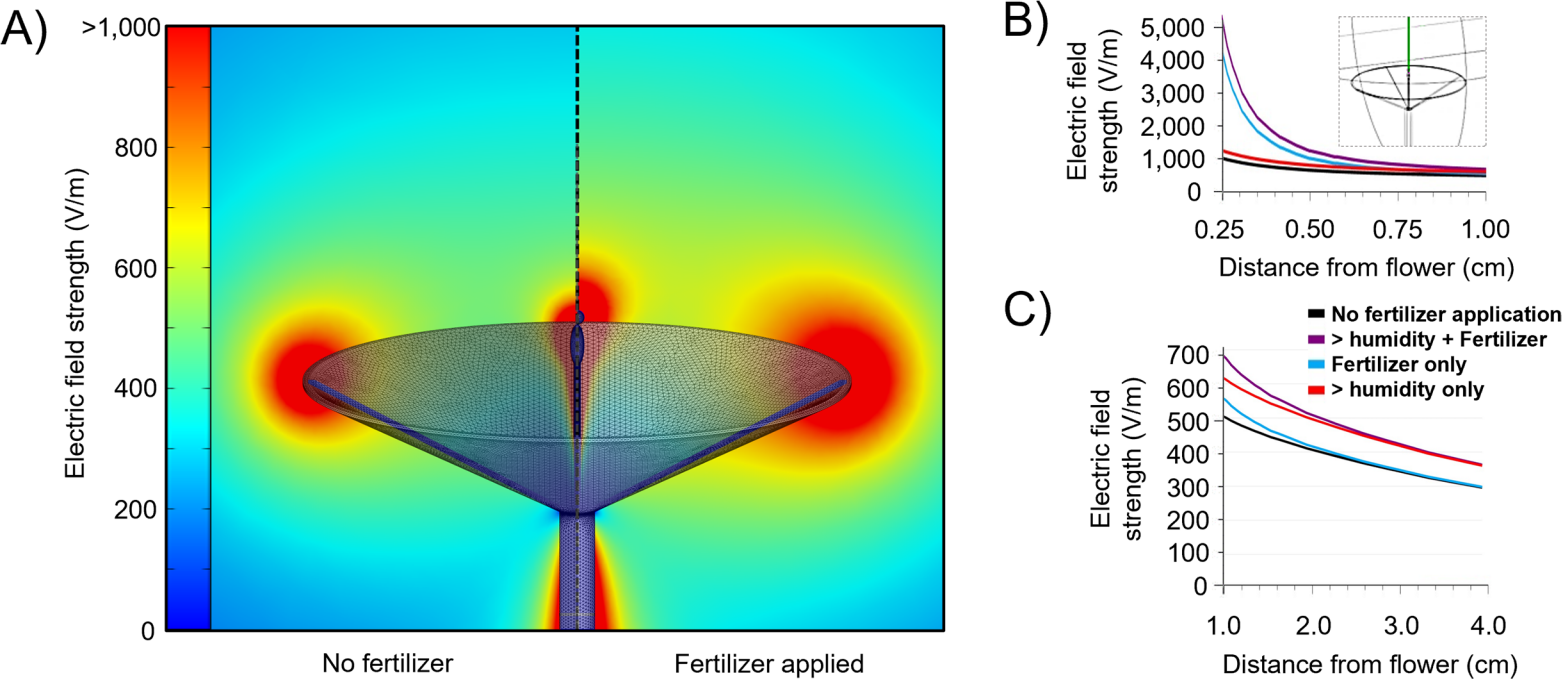
A three-dimensional finite element model depicting the influence of fertilizer application upon a flower exposed to a fair-weather atmospheric potential gradient. (A) Side-by-side comparison of 2D slices through the center of the flower (3D model superimposed on model output), untreated flowers (left), and flowers treated with a fertilizer spray application (right) in a 100 V/m atmospheric E-field, in which color gradients indicate E-field strength. Please note that floral E-fields above 1,000 V/m are grouped as > 1,000 for clarity. (B and C) Electric field strength values taken through the green cutline of the 3D model (inset) focusing on the most relevant distances to foraging bees, displayed for two sets of distances for clarity. Distances extend in (B) from 0.25 to 1.00 cm from the tip of the flower, and in (C) from 1.0 to 4.0 cm from the tip of the flower.

Spray applications conceivably affect a wide range of biological and physical properties relevant to flower-pollinator interactions that can involve both the plant surface (e.g. conductivity, capacitance, permittivity) as well as its surrounding air (i.e. increased humidity and decreased electrical conductivity) ( 31). The magnitude of such effects is likely dependent on applied charge and aerosol densities as well as weather conditions (e.g. windspeed). Since compound fertilizers are widely used and more frequently applied than pesticides, we centered our study on spray applications of compound fertilizers. An in-depth characterization of the effect of fertilizers on bee visitation was beyond the scope of this study, but we confirmed that a spray application of fertilizers can result in a temporary reduction in bee visitations and avoidance behavior similar as observed for other chemicals (28, 29) (Supplementary Fig. S1; Supplementary Movie S1). To yield insights into the relative magnitudes of the effect of a foliar fertilizer application on floral E-fields, we developed a finite element modeling approach. To this end, we considered two appreciable factors associated with a fertilizer spray application: the presence of fertilizer droplets upon the floral surface, and a localized increase in air humidity (see Supplementary Table S1 for parameters and their ranges). This analysis shows the marked impact of agrochemical spray application on floral E-fields (Fig.   1A to C). The presence of fertilizer droplets creates a perturbation of floral E-fields at close range, while the change in E-fields due to a decrease in air conductivity associated with increased humidity is much more pronounced at longer ranges. Combined, these factors interact to increase the magnitude of the floral E-field by as much as 40 to 500% at distances relevant for bee foraging (0.25 to 4 cm). These findings are robust within all realistic input parameter ranges (Supplementary Fig. S2). As a corollary of the direct proportionality between the bio-electric potential in the stem and floral E-fields (Supplementary Fig. S3), changes in surface wetness and air humidity will likely amplify chemical-induced changes in the floral E-field arising from alterations in the bio-electric potential. This suggests that agricultural chemicals are poised to interact with the electric landscape in multifaceted ways.

**Fig. 2.**
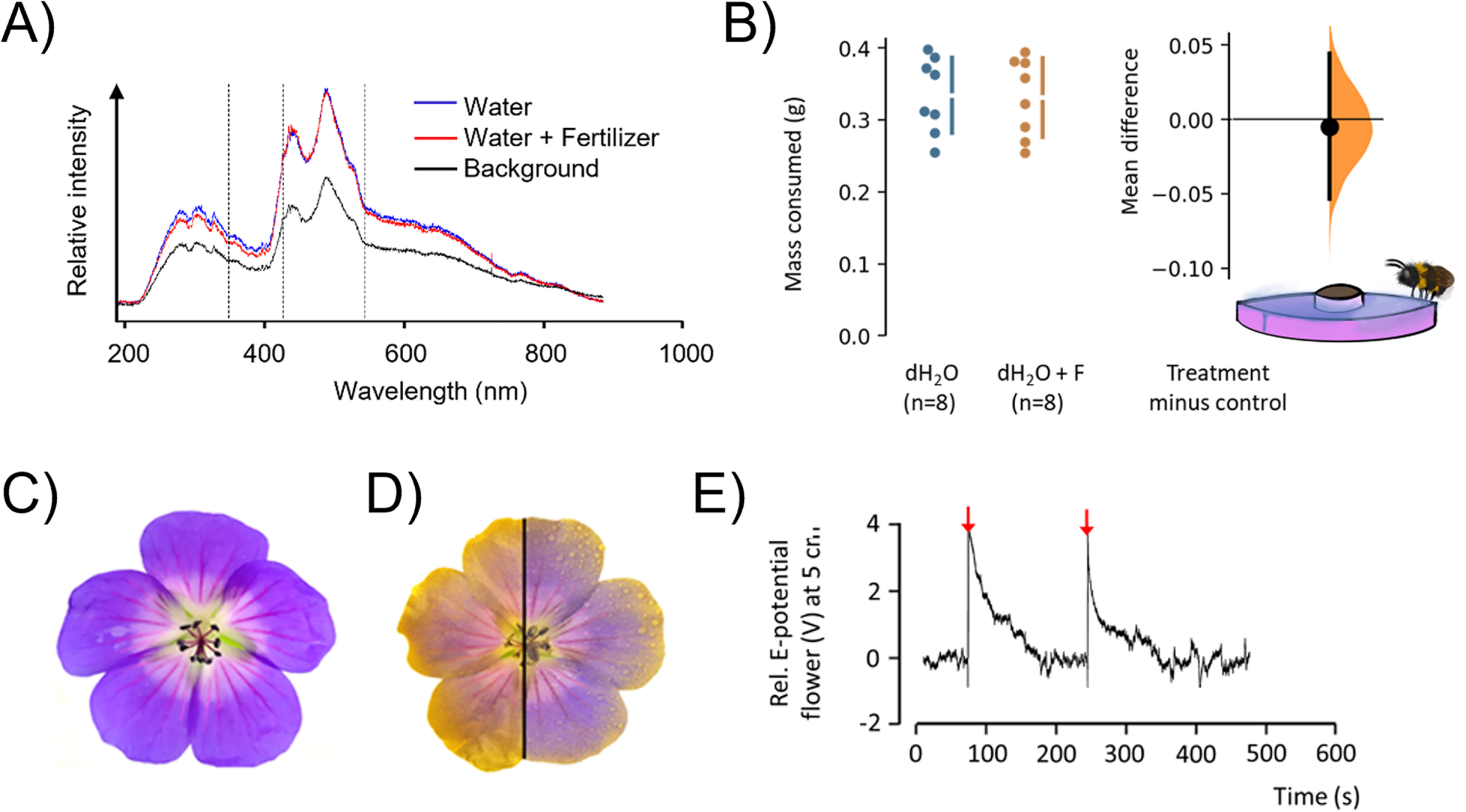
Effect of fertilizer on key floral cues used by bees. (A) Reflectance spectra for dH_2_O compared to a dH_2_O + fertilizer coating and background consisting of white paper with self-adhesive film. Dashed vertical lines represent *B. terrestrisdalmatinus* photoreceptor spectral sensitivity peaks: 348, 435, and 533 nm (30). (B) Repellence of fertilizer was assessed by measuring feeding effort in grams of sucrose consumed per 2 hours using feeding disks where surfaces were wetted with dH2O or dH_2_O with fertilizer (dH_2_O + F). Unpaired mean difference between dH_2_O and dH_2_O + F is −0.00475 [95%CI −0.0532, 0.0443], *P* = 0.85; two-sided permutation *t*-test. (C and D) Representative example (*n* = 20) of flowers (*G. magnificum*) before (C) and after (D) spraying with electrostatic colored powder without a fertilizer spray application (left half) and with a fertilizer spray application (right half). The pattern of powder deposition reflects the variation in E-field strength at the flowers’ surface. (E) Effect of fertilizer spray application on the relative electric potential of Ragwort (*J. vulgaris*), at 5 cm distance from the flower. Arrows indicate the time of fertilizer treatment.

As bees rely on multimodal signaling whilst foraging, various floral cues could be affected by agrochemical spray application. For example, fertilizers could potentially affect visual cues by altering the spectral properties (32) of flowers and thereby misguide bees. To test this, the reflectance spectra of spray applications with demi-water (dH_2_O) containing realistic fertilizer concentrations (5 mL/L^−1^; F-dH _2_O) and a control dH_2_O-only application were measured on a background of white paper with self-adhesive covering film. This spectrophotometric test revealed no difference between treatments (Fig. 2A), suggesting no visual difference exists between treated and untreated flowers. Moreover, odor can modulate foraging as bumblebees can discriminate flower and pollen odor ( 33, 34). While fertilizers used here are not volatile under these conditions (boiling point >300 °C), we tested the potential olfactory repellence of fertilizers by offering sucrose-only solutions to bumblebees ( *Bombus terrestris*) in purple molds in laboratory flight arenas where the surrounding feeding platforms were sprayed F-dH _2_O (5 mL/L^−1^). Sucrose consumption was quantified relative to control feeding platforms sprayed with dH_2_O-only (see “Supplementary Material” for details). It has been previously shown that the wetness of plant surfaces does not affect bumblebee foraging behavior (35). In this experiment, we observed that bumblebees were actively feeding on both treatments, resulting in no detectable differences in consumption rates between treatments (Fig.   2B), confirming that fertilizers do not alter the odor of the food source. To explore whether chemical treatments have the potential to affect floral E-fields, we sprayed cut flowers (*Geranium pratense*) with F-dH_2_O and subsequently coated the flowers with positively charged, colored particles released as an aerosol close to the corolla. The electrostatic deposition pattern of colored particles consistently differed between untreated flowers and flowers treated with F-dH_2_O (*n *= 20, representative example: Fig. 2C and D). Although we cannot exclude a possible electrostatic interaction between fertilizers and charged color particles, these results suggest that a fertilizer application alters the structure of floral E-fields. To assess whether such changes can be observed in natural plants rooted in soil, we measured the E-field at a 5 cm distance from a flower (*Jacobaea vulgaris*) in response to a fertilizer spray application. Although a measurement closer to the flower is required to capture the true magnitude of floral E-field change, this measurement revealed that spray applications of fertilizers can instantly increase floral E-fields followed by a slow decay over the course of several minutes (Fig.   2E). Collectively, this suggests that fertilizers are not visually and olfactory repellent, but that they have the potential to elicit structural and dynamic changes in floral E-fields.

**Fig. 3.**
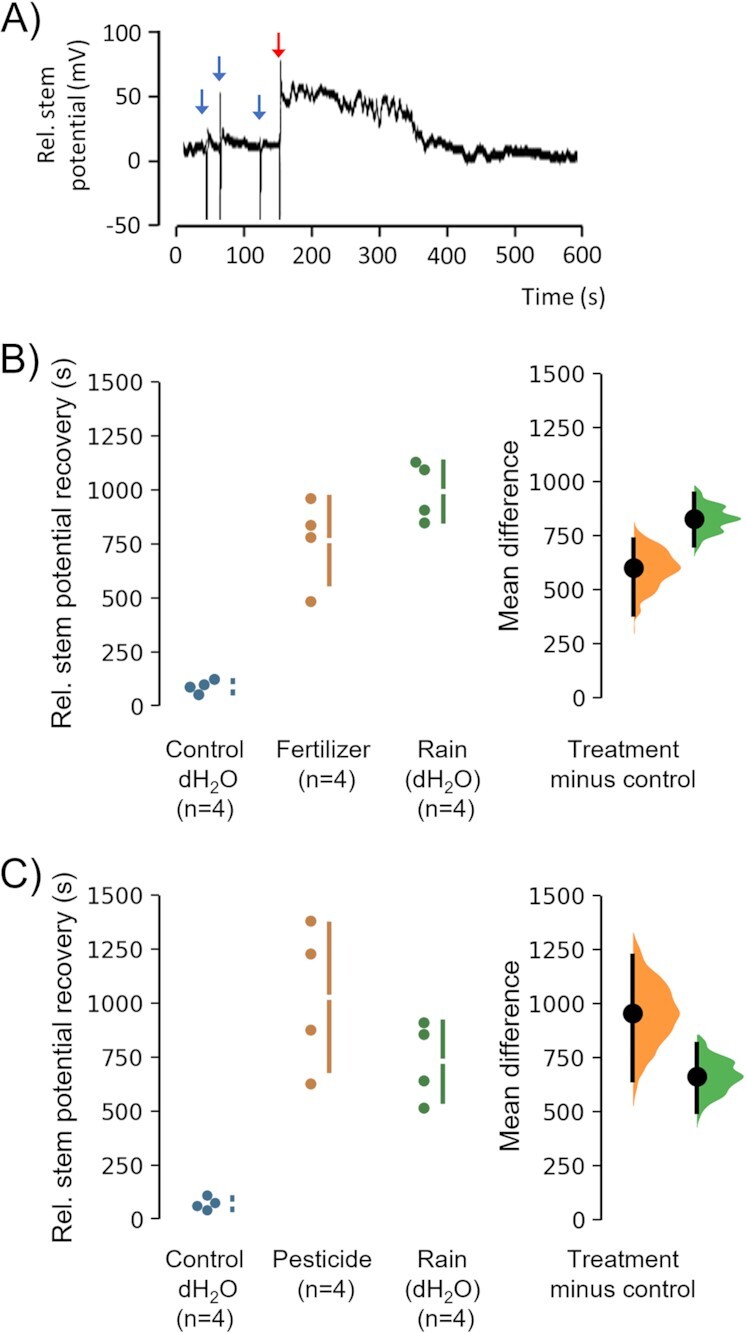
Effect of chemicals on stem potential. (A) Dynamic change in the relative stem potential of cut lavender (*L. angustifolia*) after a spray application with dH_2_O (blue arrows) or dH_2_O containing fertilizers (red arrow). The effect of fertilizer (B) and imidacloprid (C) deposition on the stem streaming potential of *E. russelianum* as reflected by the time required for recovery of stem potential after spray events of either a control dH_2_O (blue) or chemical treatment (orange). A second spray of dH_2_O was applied after the chemical treatments to mimic a subsequent rain event (green). Unpaired mean difference between Control and Fertilizer is 674 [95%CI 460, 840; P<0.001], and Rain is 940 [95%CI 780, 1020; P<0.001]. Unpaired mean difference between Control and Imidacloprid is 979 [95%CI 664, 128; P<0.001] and Rain is 654 [95%CI 488, 802; P<0.001].

The temporary nature of observed foraging declines (28, 29; Supplementary Fig. S1; Supplementary Movie, S1) suggests dynamic changes in floral E-fields are potentially relevant. Such changes in floral E-fields reflect a composite change in plant physiology and the physical properties of both the plant and the surrounding air, many of which are unknown or difficult to measure and to control. A known physiological response of plants to external stressors (e.g. herbivory, chemicals, and acid rain) is the alteration in bio-electric potential within the stem, where a change in water flow and ion transport is visible as a change in potential difference or streaming current (36–39). Changes in stem potential can be measured and manipulated and are directly proportional to E-field strengths near the flower (Supplementary Fig. S2), and therefore used here to explore the significance of chemical-induced floral E-field changes for plant-bee interactions. In laboratory recordings, we measured temporal changes in the relative stem potential generated by an ion flux that is proportional to the streaming potential (5, 37). We observed that the deposition of fertilizers substantially alters the stem potential of cut *Lavandula angustifolia* flowers for prolonged periods (Fig. 3A; Supplementary Fig. S4). To accurately determine the extent to which fertilizers alter plant bio-electric potentials, we measured stem potential changes in larger and easier-to-handle stems of *Eustoma russellianum* in response to sprays with control dH_2_O and dH_2_O-containing fertilizers. While we observed a change in stem potential induced by a control dH_2_O spray application that only lasted 30 to 60 seconds (Fig. 3B), a spray with fertilizers consistently resulted in a change in stem potential that lasted up to 16 min. To test whether similar responses could be induced by another agricultural chemical, we used a spray application with the positively charged imidacloprid, an emblematic neonicotinoid pesticide. Here, imadocloprid induced a change in stem potential that lasted up to 25 min (Fig. 3C). These changes did not appear to be the result of surface wetness as flower surfaces remained wet for prolonged periods (>1 hour) after spray applications. The observed fluctuation in stem potential upon agricultural spray application is up to 15 times longer than natural fluctuations (e.g. wind or bees, ∼100 seconds) (14, 27), and aligns with observed declines (approximately 20 min) in foraging after spray applications in natural field conditions (28, 29) (Supplementary Fig. 1). Since many chemicals used in agriculture and horticulture carry an electric charge, the observed mechanism could potentially be relevant for a wide array of chemicals.

To further assess how the treated plants would respond to a subsequent rain event, we mimicked a rain event after the initial treatment by spraying dH_2_O considering rain in clean areas is usually free from geogenic chemicals (40). Again, a prolonged alteration of the electrical signature that corresponds to the chemical treatments was also observed after the simulated rain event (Fig. 3B and C). This unveils recurrent effects of topical chemical deposition or imprinting on plant physiological responses. While the long-term effects of the observed chemically induced changes in floral E-fields and pollinator-plant interactions remain uncertain, it is conceivable that the adverse effects of spray applications can persist in time due to (1) recurring changes in the bio-electric potential following rain events, and (2) a lasting negative association learned by visiting bees or observing conspecifics (41–43).

**Fig. 4.**
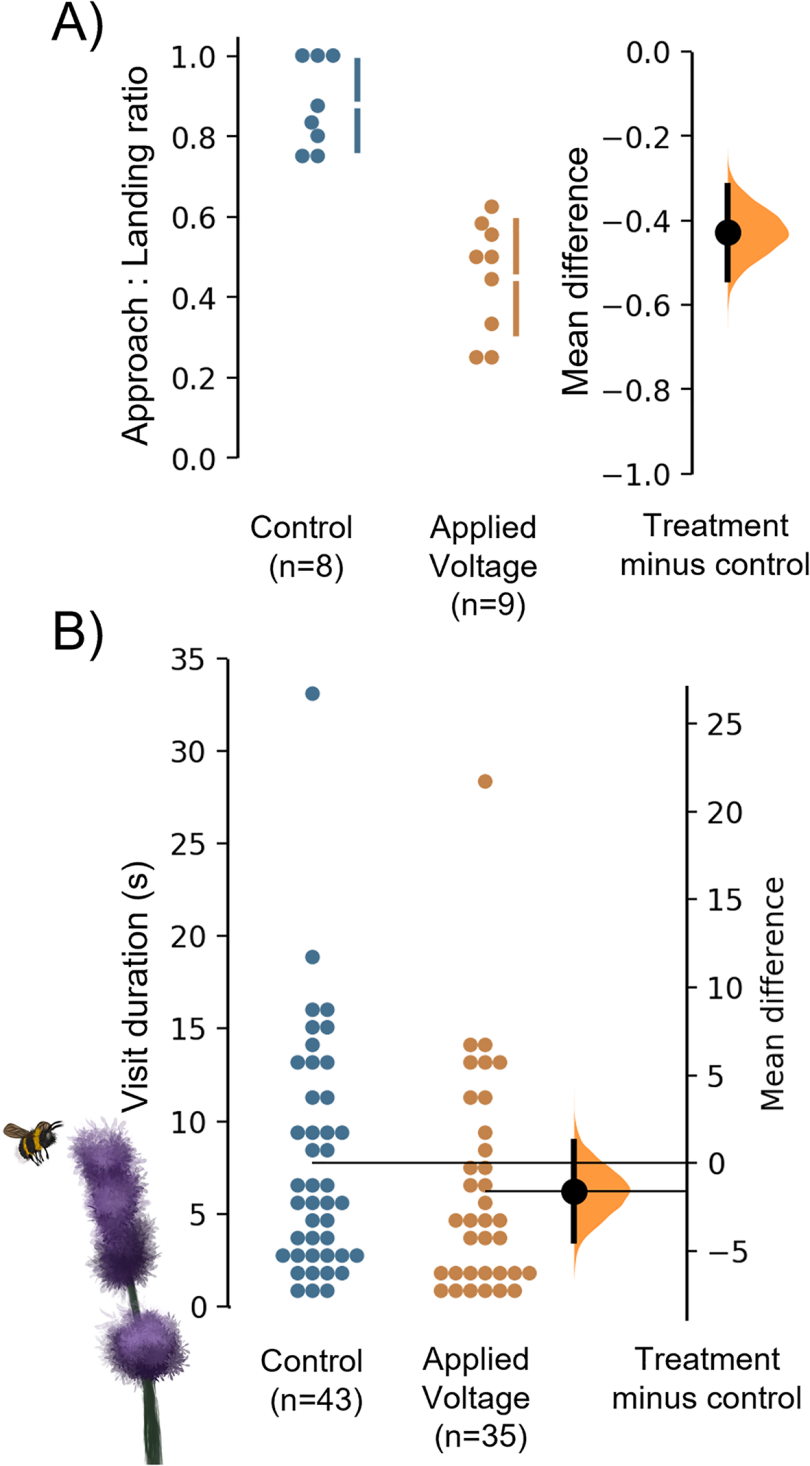
Foraging behavior of wild bumblebees on control lavender flowers and lavender flowers where stems were artificially maintained at a different electric potential. Stem potential was calibrated against changes observed after spray applications of fertilizer (approximately 500 mV induced by a 13 V potential difference across the flower and lower end of the stem). (A) Ratio between bee approaches and landing over the replicate runs. The total number of approaches was 47 for control and 62 for applied voltage, while the total number of landings was 43 for control and 35 for applied voltage Unpaired mean difference between Control and manipulated flowers is -0.427 [95%CI -0.54, -0.319; P<0.01]. (B) Visit duration of landed bees. Unpaired mean difference between control and manipulated flowers is −1.62 [95%CI −4.44, 1.17; P=0.285].

Fertilizers and a potentially wide array of agricultural chemicals can thus affect the electrical signature of flowers, likely affecting how it is perceived by bees. To assess this, we observed foraging behavior of wild bumblebees on cut *L. angustifolia* flowers in a rural area in which we artificially maintained the flower stems at a different potential to mimic the bio-electric changes induced by agrochemical spray applications. The change in potential was calibrated against changes observed upon a fertilizer spray application (approximately 500 mV; see Fig. 4A), in which we observed that a 13 V potential difference across the flower and stem resulted in a change in streaming current that is comparable to changes due to fertilizer sprays (see “Supplementary Material”). The signal generator was set at an 0.07 Hz square wave to maintain a direct current (DC) component yet prevent drift of the stem potential offset. The total number of approaches was 47 for control flowers and 62 for manipulated flowers, while the total number of landings was 43 for control flowers and 35 for manipulated flowers (average approaches and landings per run: control 5.9 ± 3.4 and 4.8 ± 3.1 and manipulated flowers: 7.8 ± 3.4 and 3.6 ± 2.1, respectively.). Quantifying bee approach to landing ratio shows that flowers with altered stem potential and floral E-fields receive significantly fewer landings from bees compared to control flowers without manipulated stem potentials (Fig. 4A). To assess whether electric manipulation of the flowers affected bees on the flower, we measured the visit duration of landed bees (Fig. 4B), revealing no apparent difference between treatments. This suggests altered floral E-fields affect bee foraging when approaching the flower, and that bumblebees can detect and discriminate small and dynamic alterations in the electric landscape induced by agrochemical deposition. This encourages investigation of chemical-borne perturbations in the electric landscape and whether this affects the health and performance of pollinator populations.

The present evidence that fertilizers affect pollinator behavior by interfering with the way an organism perceives its physical environment offers the first insights into the effects of human-made chemicals on the biologically relevant electrical environment. These effects are potentially also induced by various airborne particles (e.g. nanoparticles, exhaust gases, nanoplastics, and viral particles). Although it remains speculative how electric alterations operate at larger spatial and temporal scales, the potential of human-made chemicals to alter a complex set of interlinked biophysical processes likely carries implications beyond pollination since natural E-fields are pervasive and intrinsically linked to a wide array of organisms and biological processes (7, 44–49).

## Supplementary Material

pgac230_Supplemental_Files

## Data Availability

All data are included in the manuscript and/or supporting information.
