# Supplementary material for: Synthetic fertilizers alter floral biophysical cues and bumblebee foraging behavior
Source: PNAS Nexus. 2022 Nov 9;1(5):pgac230. doi: 10.1093/pnasnexus/pgac230 (PMC9802097; doi:10.1093/pnasnexus/pgac230)
Supplement: pgac230_Supplemental_Files [file pgac230_supplemental_files.zip › PNASNEXUS-PNASNEXUS-2022-00498-T-s01.docx]

**Supplementary material**

Synthetic fertilizers alter floral biophysical cues and bumblebee foraging behavior

Ellard R. Hunting^1*^, Sam J. England^1^, Kuang Koh^1^, Dave A. Lawson^1^, Nadja R. Brun^2†^, Daniel Robert^1^

*^1^ School of Biological Sciences, University of Bristol, Bristol, United Kingdom*

*^2^ Biology Department, Woods Hole Oceanographic Institution, Woods Hole, MA, United States*

† *Present address:* *Department of Biological Sciences, University of Bergen, Bergen, Norway*

Ellard R. Hunting:  [e.r.hunting@bristol.ac.uk](mailto:e.r.hunting@bristol.ac.uk)

1. **Materials and Methods**

***Modelling - Finite element analysis***

Computational modelling of the effect of fertilizer application on floral electric fields was performed using finite element analysis in COMSOL Multiphysics® v. 5.4 (COMSOL AB, Stockholm, Sweden) equipped with the ‘Electric Currents’ interface within the ‘AC/DC’ module. The geometry of the model consists of a 2 x 2 x 1 m (width x depth x height) cuboid in which the rest of the geometry was contained. The top surface of this cuboid is held at a 100 V potential, whilst the bottom surface is defined as the electrical ground to create a vertical potential gradient equivalent to 100 V/m, typical of the atmospheric potential gradient in fair-weather conditions (Wilson 1903; Bennett and Harrison 2007). The flower is located at the center of the cuboid, attached to the grounded bottom surface. The flower geometry was comprised of a conical structure with a height of 20 mm, a radius of 40 mm, and a thickness of 1 mm, representing the petals. Within this lies an ellipsoid with 1 x 1 x 3 mm semi-axes, mounted upon a 25 mm tall, 1 mm diameter cylinder, altogether representative of a stamen. This flower head then sits atop a 30 cm tall stem with a diameter of 3 mm. In the variation of the model in which fertilizer has been applied to the flower, a 2 mm diameter sphere is located at the tip of the stamen, representing a droplet of fertilizer, as well as a 20 cm diameter sphere surrounding the flower, representing a region of increased humidity caused by a spray of fertilizer. Meshing of this geometry was user-controlled, with a minimum element size of 0.0001 m and a maximum element size of 0.04 m. The parameters for the electrical properties of each material, are provided Table S1, along with the sources of this information. Parametric sweeps were also performed for all these values to check the model output for parameter selection sensitivity. Sweeps were also performed on the strength of the vertical atmospheric potential gradient applied, as well as the strength of a potential applied to the base of the flower head (to investigate the impact of stem potential changes on the floral electric field with APG both present and absent). All other parameters were held constant at their primary model values whilst sweeping a particular parameter. The ranges of parameter values used in all these sweeps are also provided in Table S1. The results of these parameter sweeps show that the model output is robust within realistic value ranges for all parameters, see figure S2.

**Table S1:** *Parameters and their ranges used for the computational modelling of the effect of fertilizer application on floral electric fields using finite element analysis in COMSOL Multiphysics^®^.*

| **Parameter** | **Value** | **Parametric sweep range** | **Source** |
| --- | --- | --- | --- |
| Air conductivity | 1 x 10^-14^ S/m | 1 x 10^-16 –^ 1 x 10^-13^ S/m | Hogg 1939; Higazi and Chalmers, 1966 |
| Air relative permittivity | 1 | N/A (not thought to vary substantially) | Well known quantity |
| Humid air conductivity | 1 x 10^-15^ S/m | 1 x 10^-16 –^ 1 x 10^-13^ S/m | Moore and Vonnegut (1988); Kamra et al. (1997); Harrison and Aplin 2007 |
| Humid air relative permittivity | 1 | 1 – 2 | Well known quantity |
| Plant conductivity | 0.1 S/m | 1 x 10^-10^ – 1 S/m | Nadler et al. 2008; Jeon et al. 2018. |
| Plant relative permittivity | 35 | 2 – 80 | Ulaby and Jedlicka 1984; Dadshani et al. 2015 |
| Fertilizer conductivity | 0.0923 S/m | 1 x 10^-5^ – 10 S/m | Measured in laboratory; average of ten measurements with Hach Multi-probe submerged in BabyBio plant food solution (SBM Life Science Ltd., Cambridge, UK) prepared as per manufacturers instructions with tap water (average conductivity 0.0537 S/m) |
| Fertilizer relative permittivity | 80 | 1 – 80 | Well-known value of water |
| APG Strength | 100 V/m | 0 – 2500 V/m | Wilson 1903; Bennett and Harrison 2007 |
| Stem potential | Not present in primary models | -0.5 – 0.5 V | Clarke et al. 2013 |

***Experimental***

*Chemicals*

The most commonly applied fertilizers in horticulture, gardening and increasingly agriculture are compound fluid fertilizers composed of a mixture of nitrogen (N_2_), Phosphorus Pentoxide (P_2_O_5_), Potassium Oxide (K_2_O), in which N:P:K ratios can differ depending on purpose. Throughout this study, we used a commercially available compound fluid fertilizer (Nitrogen, Phosphorus Pentoxide, Potassium Oxide, 10.6:4.4:1.7; Bayer, Leverkusen, Germany) following manufactures instructions (5mL/L). Neonicotinoid insecticide imidacloprid (99.7% purity, CAS Number: 138261-41-3) purchased from Sigma-Aldrich (Zwijndrecht, The Netherlands). Nominal concentration of imidacloprid was 3.5 μg/L dissolved in demineralized water in glass bottles (Solubility: 610 mg/l H_2_O at 20 °C). Throughout the study, chemical were applied using a single spray with a commercially available gardening spray bottle.

An in-depth characterization of the effect of fertilizers on bee foraging behavior was beyond the scope of this study, but a small behavioural experiment was performed to confirm that a spray application of fertilizers can result in a temporary reduction in bee visitations and avoidance behavior similar as observed for other chemicals (e.g., Fell et al., 1983; Desneux et al., 2007). To this end, foraging behavior of a natural population was assessed at a field site on the campus of the School of Veterinary Sciences of the University of Bristol, Langford UK, in the summer of 2018. This site is positioned in a rural area. Foraging of bumblebees was monitored by quantifying bee visits using two potted Lavender plants (*Lavandula* sp.) on fair weather days without wind during foraging peaks (between 11:00 and 14:00). To this end, bee visits were counted for 12 minutes to obtain a baseline. After 12 minutes, one potted *Lavandula* sp. was sprayed with demineralized water containing common compound fluid fertilizer following manufactures instructions (5mL/L) while a control treatment was sprayed with demineralized water. This was repeated three times.  This confirmed that spray applications with fertilizer can indeed result in a short-term reduction of bee visits (Suppl. Fig. 1).

*Spectral comparison*

Reflectance spectra of demineralized water and demineralized water containing compound fertilizer as used throughout the study were measured using an Ocean Optics Flame-S- UV-VIS miniature spectrometer (Ocean Optics, Florida, US). A deuterium - halogen tungsten lamp (DH-2000-BAL UV-VIS-NIR; Ocean Optics, Florida, US) was used as a standardised source of light, with measurements taken using a premium-grade reflection probe (QR400-7-UV-VIS; Ocean Optics, Florida, US). The axis of the illuminating and reflection probe was perpendicular (90°) to the sample plane. The spectrometer was calibrated using a white spectralon standard (WS-1-SL, Ocean Optics, Florida, US).

*Chemical repellence*

Individual compounds as well as their dissolution and degradation products are not volatile (i.e., boiling points > 300^o^C) under the physico-chemical conditions of the experimental set up. To rule out potential chemical repellence, feeding assays were performed using a flower and fertilizer naïve bumblebee (*Bombus terrestris*) colony (Kopperts, NL). The colony is housed in a plastic nesting box which was connected to a common flight arena (72 x 104 x 30 cm) divided over four compartments. Bees accessed the flight arena from the colony via a tube to prevent stress, regulated by a series of manually controlled gates. Four bees were allowed to access each compartment before closing. Bees were subsequently offered two purple feeding disks that both contained a 30% glucose solution, but where the purple disk was sprayed with either demi-water or demi-water containing common compound fluid fertilizer following manufactures instructions. Each run lasted 2 hours, and the mass of glucose consumed was quantified by determining mass difference before and after trials. Feeding disks were rinsed with 70% ethanol between trials, and each feeding experiment was replicated eight times.

*Floral electric field*

Changes in E-fields can be visualized using a non-quantitative spraying of charged paint particles. To visually assess whether fertilizer treatments affect floral E-fields, we sprayed flowers with F-dH_2_O and subsequently coated the flowers with positively charged, colored particles released as an aerosol close to the corolla. For this experiment we used *Geranium pratense* as they are geometrically simple and therefore allow for an easier detection of changes in particle deposition patterns. The electrostatic powder coating system used was manufactured by ElectrostaticMagic™ (Nottingham, UK). Fine particles (ca. 30-50µm diameter) of coloured plastic are given a large positive charge by a high-voltage electrode and then aerosolized in the vicinity of the flower by means of a compressed air supply. The plant is grounded with an inserted electrode at the base of its stem, mimicking the ground connection the plant would have naturally in the soil. Electrostatic powder was applied to untreated flowers and flowers sprayed with demi water containing fertilizer. The flowers were photographed immediately after a small application of the powder coat. Areas of high powder density on the flower are areas where the local electric field is strongest. The procedure was repeated 20 times.

The changes in floral electric field resulting from application of electric potentials to the plant stem were measured with a bespoke electrometer. To assess the relationship between stem potential changes and floral electric fields, a calibration curve was produced using cut inflorescence of *Lavandula* sp. positioned with its stem in a glass jar with tap water, 40 cm above the ground. A Koolertron DDS signal generator (Shenzhen Kuletong Technology Co. Limited, Shenzhen, China) was connected electrically to the plant, with one output connected to a tungsten electrode piercing the stem. The other output was connected to the water. Sine wave signals with a frequency of 0.2Hz were generated at peak-to-peak voltages decreasing in increments of 2V, from 18V to 2V. A 1cm diameter steel ball electrode was mounted at the end of a wooden handle, held within a clamp stand. The signal wire for the electrode was shielded and connected via a triaxial cable to the electrometer. This electrode was placed at 1cm distance from the *Lavandula* sp. flower-proper to measure the changes in the floral potential generated by potentials applied to the stem. The output of the electrometer was recorded with sampling frequency of 1000Hz using a NI USB-6001 DAQ device (National Instruments Corp., Austin, Texas, U.S.A) connected to a laptop acquiring the data in MATLAB (R2018a). All measurements were made in open ground in fair weather conditions. For each voltage amplitude applied to the stem, the peak-to-peak change in floral potential (V_pp_) as measured by the electrometer at 1cm was calculated for 10 full cycles of the signal and then averaged. Plotting the mean V_pp_ measured by the electrometer at 1cm from the flower against the corresponding voltage applied to the stem yielded a strongly linear relationship (Figure S2, R^2^ = 0.998, p<0.0001). The measuring electrode did not pick up a signal when the flower was subsequently cut just above the electrode, revealing that the measured signal was indeed originating from the plant. This setup was subsequently used to measure electric field changes surrounding a flower in response to a fertilizer spray application. For this experiment, we used a ragwort plant (*Jacobaea vulgaris*) because they have large singular protruding flower heads. An electrode was mounted on a tripod and positioned 5cm from the center of a flowerhead that was approximately 0.5m tall. Although it would be necessary to position the electrode as close as possible to the flower head to capture the true magnitude of floral E-fields, we positioned the electrode at 5cm distance to prevent spray droplets landing on the electrode. A spray bottle fixed in place by a second tripod was placed roughly 10cm from the plant but at such an angle that the electrode would not be impacted by the spray. A fertilizer spray was applied twice several minutes apart during the measurement window. Preliminary runs showed that the electrometer was not responding to spray applications in the absence of the flower, suggesting observed dynamics where the results of changes in the electrical properties and humidity around flowers.

*Plant electrophysiology*

A known bio-electrochemical response to external stimuli is a change in number of ions transported within the stem, measurable as a change in current that is proportional to the streaming potential (Gindl et al., 1999; Xue et al., 2017). These currents were measured in a Faraday cage in a stem of cut *Lavandula* sp. using a tungsten electrode inserted into the stem 5 cm below the petiole following methods outlined by Stankovic and Davies (1996). The electrode is connected to a WPI DAM-50 high-impedance differential amplifier (World Precision Instruments, Sarasota, Florida) in a single-ended configuration. The change in streaming current in response to different spray applications was recorded onto a PC using a National Instruments (Austin, Texas) DAQ system. Recordings on a control aluminium stick were performed to assess electrical variation induced by the spray application itself, which revealed only short variations (<5 seconds), confirming the electrical recordings are governed by plant electrophysiological responses to treatment (Fig S3). Recordings of stem streaming currents were subsequently repeated with *Eustoma russellianum* as these flowers are larger and easier to handle. Stem streaming current was allowed to reach a baseline current after an initial wounding response induced by cutting of the stem. Stem streaming current was subsequently monitored for an experimental sequence of spray applications. The sequence started with three times control spray in with demineralized water to ensure a proper connection of the electrode and electrophysiological response of the flower. Successful sequencessubsequently received a spray application with fertilizer. To assess whether the physiological response of the plant to the treatment persisted after the treatment, a subsequent spray with demineralized water was applied to mimick a subsequent rain event. Each spray was applied once stem current was recovered, in which it should be noted that flowers were still wet from the previous spray. This was replicated in four biological specimens. This exact approach was also used to assess the effect of a spray application of the common neonicotinoid insecticide imidacloprid (99.7% purity, CAS Number: 138261-41-3) purchased from Sigma-Aldrich (Zwijndrecht, The Netherlands). Nominal concentration of imidacloprid was 3.5 μg/L dissolved in demineralized water in glass bottles (Solubility: 610 mg/l H_2_O at 20 °C), applied using the same spray bottles as the fertilizer treatment. The time needed for the stem streaming current to recover to its initial baseline current after each spray application was subsequently determined.

*Foraging behavior in response to manipulated E-field changes*

Effect of manipulated changes in floral E-fields on foraging behavior of natural bumblebees was assessed in a rural area (Mendip, Somerset, UK), in the summer of 2020. To this end, two cut inflorescences of *Lavandula* sp. were positioned with their stem in a glass jar with tap water, 1 meter apart and 40 cm above the ground, in which both flowers had a tungsten electrode piercing the stem. One flower served as control, while in the other changes in stem streaming current were mimicked using a portable DC-battery operated Koolertron DDS signal generator (Shenzhen Kuletong Technology Co. Limited, Shenzhen, China) connected electrically with one output connected to the tungsten electrode piercing the stem and the other output connected to the water. This connection was calibrated against a fertilizer spray application in which stem streaming currents were measured in a Faraday cage in stems of cut *Lavandula* sp. as described above. To this end, the flower received a fertilizer spray application, and subsequently the stem steaming current was manipulated by increasing the amplitude of the signal generator to reach a change in streaming current that is comparable to the spray application. This revealed a relatively high ohmic resistance as previously observed in plants (e.g., Milner & Chalmers, 1961; Ette 1966; Farquhar and Field, 1971; Muralimadhav et al. 1990), in which an amplitude of 12-14V mimicked a stem streaming current change elicited by a fertilizer spray application. For the experimental manipulation of floral E-fields, we therefore used a square waveform with 13V amplitude and 0.07 Hz frequency applied continuously to ensure constant amplitude and prevent drifting of the stem streaming current, thereby ensuring bumblebees were exposed to an altered floral E-field. This setup was video recorded for 2 hours (Akaso v50 fx pro), and replicated 9 times in which cut flowers were replaced for each run. For each run, the number of Bumblebee spp. approaches and landings were recorded, and its ratio calculated.

*Data analysis*

Spectral analysis and time series recordings were plotted in Matlab (2016b; R2018a), in which a notch filter was applied between 49 and 51Hz to remove any 50Hz mains noise in electrical field and electrophysiological recordings. Effects of fertilizers on bumblebee visits on *Lavandula* sp. was assessed by computing an autoregressive moving average intervention analysis on detrended bee visitation data in Past 4.0 (Hammer, 2001). Intervention was set at 12 min, and ARMA signals are presented as absolute values. Differences between treatments in the feeding and behavioral assays were assessed using Gardner-Altman estimation plots (single treatment) and Cumming estimation plots (multiple treatment) following the unpaired mean difference between control and treatments and associated effect sizes (Ho et al., 2019; [www.estimationstats.com](http://www.estimationstats.com/) ). *P-*values were derived from two-sided permutation t-tests, for which 5000 bootstrap samples were taken and the confidence interval was bias-corrected and accelerated.

*Data availability*

All data and materials produced by this study are available from the corresponding author upon request.

*References*

Bennett, A. J., and Harrison, R. G. (2007). Atmospheric Electricity in Different Weather Conditions. Weather 62, 277–283. doi:10.1002/wea.97

Dadshani, S., Kurakin, A., Amanov, S. *et al.* Non-invasive assessment of leaf water status using a dual-mode microwave resonator. *Plant Methods* **11,**8 (2015). <https://doi.org/10.1186/s13007-015-0054-x>

Desneux N. et al. (2007). The sublethal effects of pesticides on beneficial arthropods. Annu. Rev. Entomol. 52, 81-106 .

Ette, A. I. I. (1966). Measurement of electrode by-passing efficiency in living trees. Journal of Atmospheric and Terrestrial Physics, 28(3), 295-302.

Farquhar, G. D., & Field, C. D. (1971). Transpiration-linked Short-circuit Currents in the Xylem of a Liana. Journal of Experimental Botany, 22(4), 818-829.

Fell R.D. et al. (1983). Effects of fungicide sprays during apple bloom on pollen viability and honeybee foraging. Environmental entomology 12(5) 1572-1575.

Harrison, R.G., Aplin, K.L. (2006). Water vapour changes and atmospheric cluster ions, Atmospheric Research, Volume 85, Issue 2, 2007, Pages 199-208, <https://doi.org/10.1016/j.atmosres.2006.12.006>.

Higazi, K. A., and Chalmers, J. A. (1966). Measurements of Atmospheric Electrical Conductivity Near the Ground. *J. Atmos. Terrestrial Phys.* 28 (3), 327–330. doi:10.1016/0021-9169(66)90042-0

Hogg, A. R. (1939). The Conduction of Electricity in the Lowest Levels of the Atmosphere. *Memoires Commonw. Observatory*. 7, 1–24.

Jeon, E., Baek, S., Choi, S., Park, K. S., & Lee, J. (2018). Real-Time Monitoring of Electroconductivity in Plants with Microscale Needle Probes. Environmental Control in Biology, 56(4), 131-135.

Joses Ho, Tayfun Tumkaya, Sameer Aryal, Hyungwon Choi, Adam Claridge-Chang. (2019). Moving beyond P values: Everyday data analysis with estimation plots. Nature Methods, 1548-7105. 10.1038/s41592-019-0470-3

Kamra, A.K., Deshpande, C.G., Gopalakrishnan V. (1997). Effect of relative humidity on the electrical conductivity of marine air Q. J. R. Meteorol. Soc., 123, 1295-130

Milner, J. W., & Chalmers, J. A. (1961). Point discharge from natural and artificial points (Pt. II). Quarterly Journal of the Royal Meteorological Society, 87(374), 592-596.

Moore, C.B., Vonnegut, B. (1988). Measurements of the electrical conductivities of air over hot water J. Atmos. Sci., 45 (5), 885-890

Muralimadhav, V., Mohanan, S., & Gnanasekaran, K. S. A. (1990). Water status of a plant body and its ohmic resistances. Bulletin of Electrochemistry, 6(05), 550-551.

Nadler, A., Raveh, E., Yermiyahu, U., Lado, M., Nasser, A., Barak, M. and Green, S. (2008). Detecting Water Stress in Trees Using Stem Electrical Conductivity Measurements. Soil Sci. Soc. Am. J., 72: 1014-1024.

Ulaby F.T., Jedlicka, R.P. (1984). Microwave Dielectric Properties of Plant Materials," in IEEE Transactions on Geoscience and Remote Sensing, vol. GE-22, no. 4, pp. 406-415. doi: 10.1109/TGRS.1984.350644.

Wilson, C. T. R. (1903). Atmospheric Electricity. Nature. 68, 102–104. doi:10.1038/068102d0.

1. ***Supplementary movie***

*Caption for supplementary Video:* Video recording of bumblebees approaching lavender flowers before and after a spray application with fertilizers. Recording shows different species and castes and was not used for comparison or quantification but shows typical hovering behavior after chemical treatment observed throughout the study.

1. **Supplementary figures**


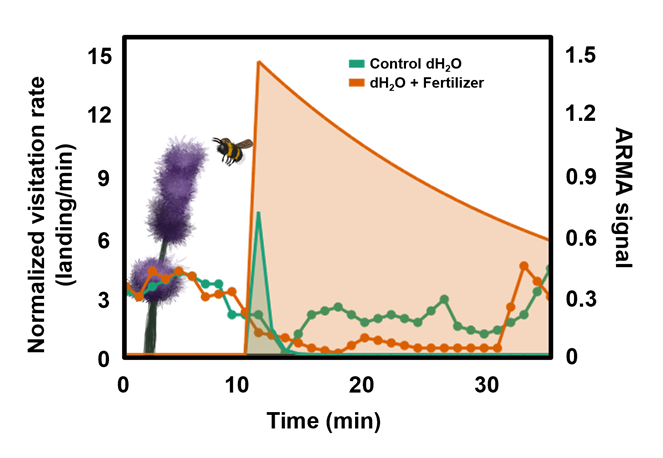


**Figure S1. Effect of compound fertilizers on bee visitation.** *Average number of flower visits per minute by bumblebees after a spray event on lavender flowers with demi water (dH_2_0 ) containing fertilizers and  dH_2_0 as a control. Spray event occurred after 12 minutes. Measurement was taken in the field based on three repeated runs. Repeats were averaged and detrended, and subsequently analyzed using an autoregressive moving average (ARMA) intervention analysis considering a pulse intervention at t=12. An ARMA signal deviating from zero points to a deviation from average visitation rates, suggesting an effect of the spray application in both treatments, with a longer lasting effect (>20 min) in the treatment containing fertilizers.*


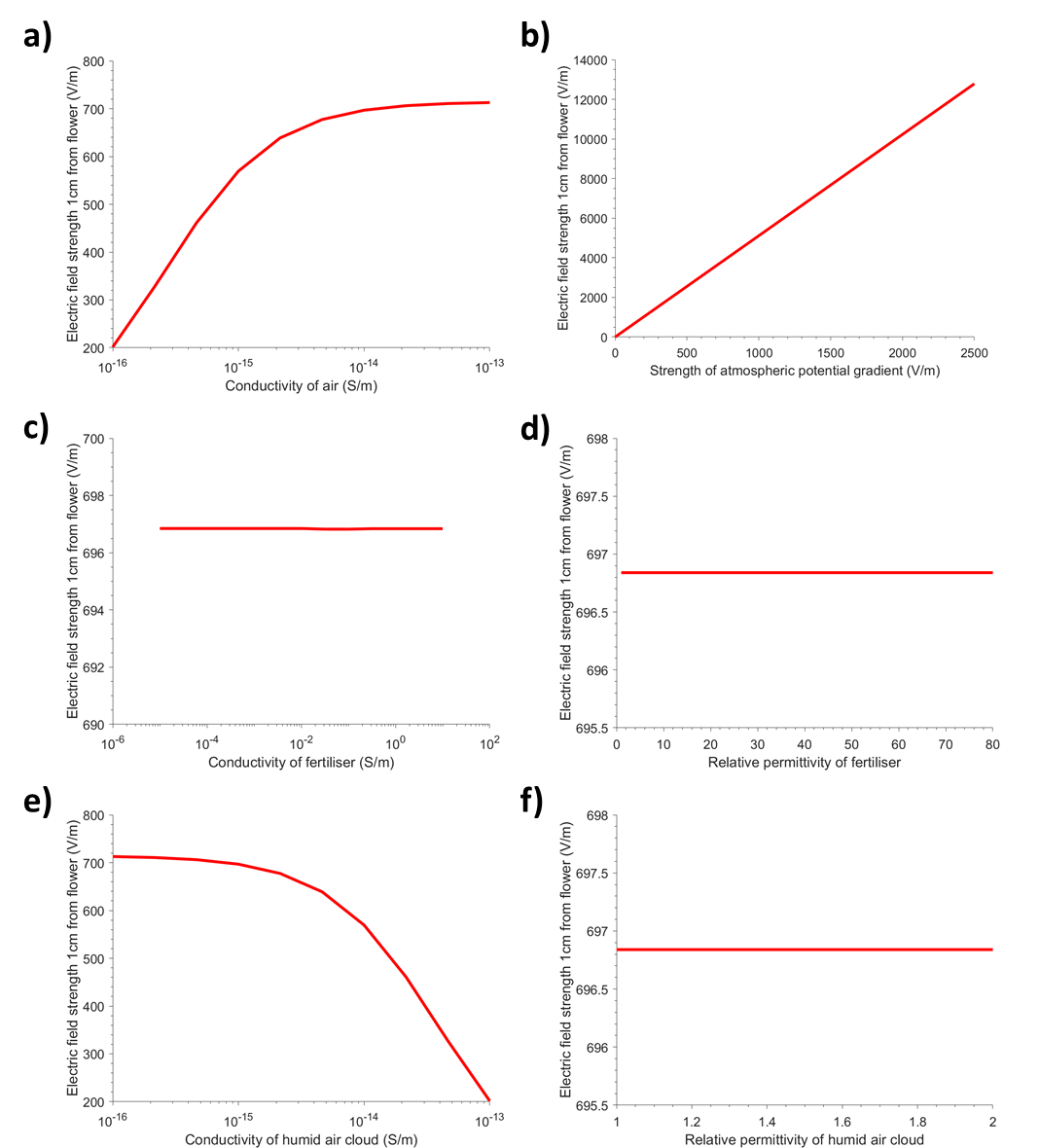
Page Break


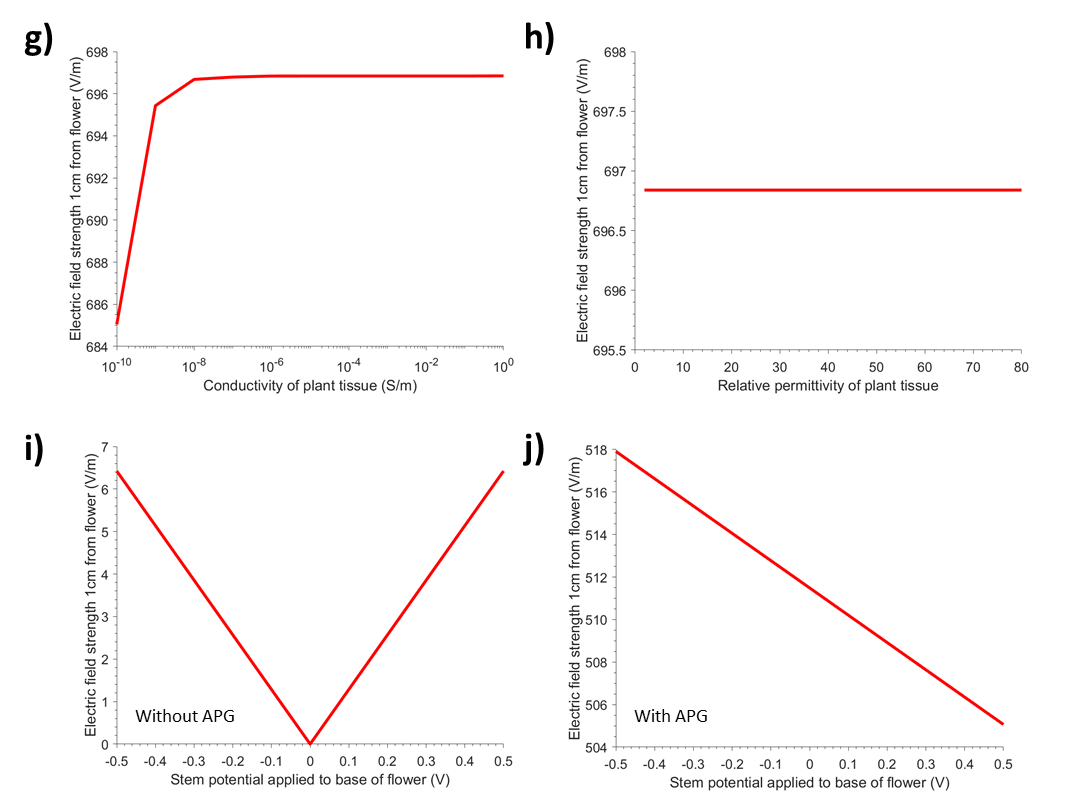


**Figure S2. Parameter sweeps for relevant parameters used in the modelling of the effect of fertilizer application on floral electric fields using finite element analysis.** *APG strength increases the floral electric field strength linearly. Applying a stem potential to the base of the flower decreases the electric field strength as the stem potential increases positively, with APG the dominant feature of the floral electric field. In a zero-APG scenario, a stem potential with either polarity increases the electric field strength. The permittivity of any of the materials including the plant has little influence, while conductivities, especially of a humid air cloud, has a strong influence.*


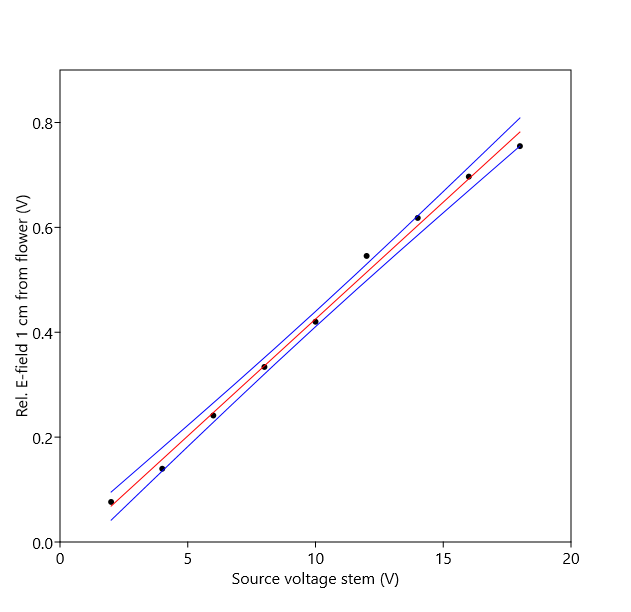


**Figure S3. Relationship between stem potential changes and floral electric fields**. *Linear relationship and corresponding 95% CL between* *mean V_pp_ measured by the electrometer at 1cm from the flower against the corresponding voltage applied to the stem (R^2^ = 0.998, p<0.0001).*


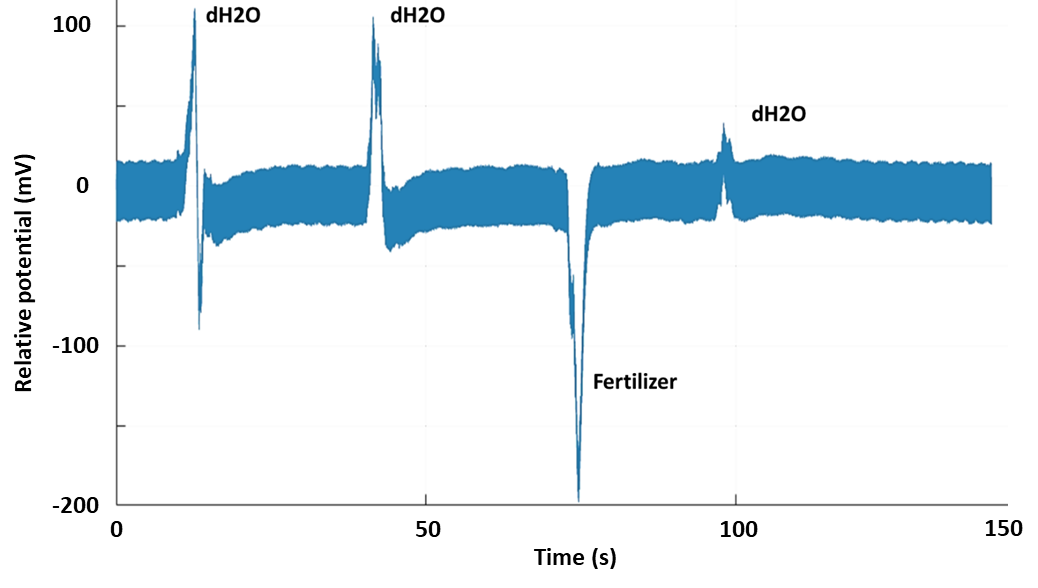


**Figure S4. Control recording of experimental setup.***Electrical recording on an aluminum plate in response to the application of demineralized water (dH20) and nutrients to assess the non-biological, electro-physical responses of the experimental set up used to assess plant electrophysiological responses. Data represents the raw recording that contains 50 Hz mains noise, in which short variations (<5 seconds) confirm the electrical recordings are governed by plant electrophysiological responses to treatment.*
